# The excitement of *Google Scholar*, the worry of *Google Print*

**DOI:** 10.1186/1742-5581-2-2

**Published:** 2005-03-22

**Authors:** Marcus A Banks

**Affiliations:** 1 Frederick L. Ehrman Medical Library, New York University Medical Center, 550 First Avenue, New York, NY USA

## Abstract

In late 2004 Google announced two major projects, the unveiling of *Google Scholar *and a major expansion of the *Google Print *digitization program. Both projects have generated discussion within the library and research communities, and *Google Print *has received significant media attention.

This commentary describes exciting educational possibilities stimulated by *Google Scholar*, and argues for caution regarding the *Google Print *project.

## Introduction

Within one month Google announced two projects that will have profound implications for the future of librarianship.

First up in November 2004 was *Google Scholar *[1]. Currently in beta, *Scholar *aims to provide access to scholarly materials via the crisp and familiar Google search box. The results pages display the number of other citations to the resource in the *Scholar *database, in a manner reminiscent of the "cited by" search feature within Thomson-ISI's *Web of Science*.

The unveiling of *Scholar *caused a flurry of excitement, and even the creation of the somewhat pretentious blog "On *Google Scholar*" [2]. At New York University Medical Center a doctor spontaneously brought up *Scholar *in conversation with me, and it also stimulated discussion at an international conference about grey literature in early December.

Soon the *Scholar *buzz was overshadowed by the December announcement that Google has entered into a partnership to digitize the materials of five leading research libraries: Harvard, Oxford, Michigan, Stanford, and the New York Public Library (NYPL). Terms of agreement vary between libraries. For example, Michigan and Stanford will provide access to the full range of their materials, while Harvard has authorized a pilot of 40,000 volumes. Depending on the copyright restrictions of the material in question, searchers will be able to browse all or part of it.

The principal rationale for this project is that it will democratize access to the intellectual resources of elite institutions. In addition, integrating library resources into Google will hopefully entice those students who might never consult a library catalog. To reach these students, Harvard plans to develop a seamless link between Google searches conducted at Harvard and Harvard's online catalog [3].

The library material represents a radical expansion of the *Google Print *program [4]. Searchers would not search for books specifically; instead, Google would highlight books within the results of a normal Google search. The library material will support the same e-commerce stream as the rest of *Google Print*. Contextual advertising would be integrated into the search results, and it is likely that searchers will be pointed to online book vendors.

Whereas the excitement about *Scholar *was concentrated in research circles, the *Google Print *projects received widespread public attention. The *New York Times *considered this to be the lead news item for December 14, and it was a major topic on the *National Public Radio *(NPR) show "Talk of the Nation" on December 15. The show's guests included Michael Keller, Stanford University Librarian, and Brewster Kahle, founder of the Internet Archive.

The Internet Archive launched a very similar digitization project as *Google Print *on the same day, which was buried in the flood of news about Google [5]. Kahle's efforts are worthy of wide promotion. His project has none of the nettlesome concerns facing *Google Print*, which I will describe later.

*Google Print *continues to generate significant discussion. One recent example is the March 2005 issue of *American Libraries*, which features a colloquium entitled, "Google at the Gate" [6]. As all librarians know, Google is the default search engine for millions of users. Because of this, it is essential that we critically examine both the benefits and shortcomings of *Google Scholar *and *Google Print*.

## Discussion

### *Google Scholar*

The initial version of *Google Scholar *had numerous flaws. Peter Jasco excellently documented these shortcomings, while promising to "write a hagiographic review about *Google Scholar *when it is done, and done well" [7]. Some of the shortcomings were that Google was unacceptably vague about its sources, and could not eliminate duplicates from search results.

Librarians must monitor the evolution of *Google Scholar*, and educate our patrons about its limits. Google's target audience for *Scholar *is "those in academia whose work has made Google itself a reality," and therefore Google aims to make *Scholar *"as useful to this community as possible" [8]. With our assistance, researchers would be able to offer suggestions about functionality that they might not consider on their own.

The educational effort about *Scholar *has already begun. The Georgia State University Libraries have developed a straightforward web page, which includes a search box for *Scholar*, the library's e-journal list, and the library catalog [9]. It is easy to foresee this page blossoming into a class about using *Scholar*, one goal of which might be to increase patron appreciation for the challenge of providing access to electronic scholarship. As patrons use *Scholar *and discover the barriers to obtaining research articles, they could be more receptive to the argument for open access publishing.

The "cited by" feature of *Scholar *presents another educational opportunity. An essential caveat that should be incorporated into this instruction is that *Scholar*'s cited by algorithms are not yet fully reliable [10]. For example, I ran a *Scholar *search for "bioinformatics," which returned "about 62,600" results within .06 seconds. I selected the third citation, which was cited by 88 other resources within *Scholar*. After 5 clicks I was down to one article, standing at the root of one chain of thematic connection.

My strategy on each screen was to click on articles that had less cited by citations than the article I selected on the screen before. Of course, I could have approached this in different ways, or tackled a different problem. The point is that *Scholar *provides such teaching opportunities, in an interface with which many people are already familiar.

A final educational opportunity I propose is a comparison of *Scholar *to products such as Elsevier's *Scirus*, which is a search engine exclusively focused on scientific research [11]. Mr. Reinhardt Wentz gave me this idea with a November 2004 posting to MEDLIB-L [12]. Unlike *Scholar*, *Scirus *differentiated a search for "bioinformatics" into journal and web page results, and also offered suggested search terms for refining these results. It also provides a "similar results" capability, which is analogous to PubMed's "related articles" feature. But it does not provide a "cited by" capability. A well-designed class would facilitate interesting discussion about the merits of these two approaches for identifying scholarly materials.

In addition to the educational work ahead, *Scholar *presents opportunities for librarian advocacy. In order to build comprehensive biomedical digital libraries, for example, it is essential that *Scholar *provides access to visual as well as textual material. Prime candidates for inclusion are the multimedia resources currently indexed in the Health Education Assets Library, or HEAL [13]. The "bioinformatics" search did not yield any results in HEAL, so I broadened it to "informatics." This had 24 results. These multimedia resources would enliven the results of a *Scholar *search, and also be placed into a broader research context than HEAL can provide on its own.

### *Google Print*

*Scholar's *lack of maturity is not surprising, because it has existed for less than 6 months. But I am hopeful that it will improve and open up new avenues for library instruction. I am less sanguine about the implications of the *Google Print *project.

At first blush I was swept up by the positive publicity surrounding the project, because it is inspiring to contemplate the democratization of knowledge that has previously been sequestered inside some of the world's leading research libraries.

After I read Rory Litwin's essay, "On Google's Monetization of Libraries," I was forced to tamper my enthusiasm [14]. Litwin argues that the e-commerce foundations of *Google Print *are antithetical to the principles of librarianship. Until now a library's resources have served as their own advertisement, but now they will become a vehicle for selling something else. And, of course, only a select group will be able to afford the items available. In its implementation, the much-heralded idea of democratization of knowledge will actually reinforce existing class distinctions.

Another concern about *Google Print*, as Litwin points out, is that it flattens the distinctions between materials that are used for different purposes. A chief reason universities select resources is because of their enduring value for scholarship; a chief reason Amazon stocks books is to make money. *Google Print *collapses this difference. My search for "gardening" might link to a priceless treatise by Linnaeus just above a link to Martha Stewart's annual review. In either case, I'll be able to order planting soil from Home Depot.

## Conclusion

My concerns with *Google Scholar *are structural, while those with *Google Print *are philosophical. Because the *Google Print *project is so enormous, it will be many years until it becomes a reality. In that time librarians should strongly advocate for ways to minimize the problems noted above. One simple solution would be to segregate the library books within search results from the other books. Assuming agreement with this suggestion, a more controversial idea would be to forbid advertising on the library results pages. If this is unacceptable, it might be possible to define acceptable categories of advertising for these pages. One model for this could be the advertising that appears on NPR and the Public Broadcasting Service.

My fear is that Google will reject such ideas, on the grounds that the library community knew what it was getting into. And Google would be right. In the admirable desire to improve access to their collections, some of our best libraries may have struck a Faustian bargain.

NOTE: All of the described searches occurred on December 23, 2004.

## List of Abbreviations Used

NYPL = New York Public Library

HEAL = Health Education Assets Library

NPR = National Public Radio

## Competing Interests

The author(s) declare that they have no competing interests.
